# Alleviation of Herbicide Toxicity in *Solanum lycopersicum* L.—An Antioxidant Stimulation Approach

**DOI:** 10.3390/plants11172261

**Published:** 2022-08-30

**Authors:** Rashid I. H. Ibrahim, Ubai A. Alkhudairi, Sultan A. S. Alhusayni

**Affiliations:** 1 Department of Biological Sciences, College of Science, King Faisal University, Al-Ahsa 31982, Saudi Arabia; 2 Department of Botany, Faculty of Science, University of Khartoum, PC 11115, Khartoum P.O. Box 321, Sudan

**Keywords:** pongamia oil, glyphosate, tomato, antioxidants

## Abstract

Application of the herbicide glyphosate in crops is a common practice among farmers around the world. Tomato is one of the crops that are treated with glyphosate to fight weed growth and loss of crop. However, tomato plants often show phytotoxic effects from glyphosate. In this study, the ability of pongamia oil derived from *Pongamia pinnata* (known also as *Millettia pinnata*) tree to alleviate the herbicide glyphosate toxicity effects in tomato (*S.* *lycopersicum* L. cv. Micro-tom) plants was tested. Tomato plants were treated with a mixture of a dose of (GLY) glyphosate (10 mg kg^−1^) and different doses of pongamia oil (PO) foliar spray (5, 10, 50, and 100 mM) and compared with the herbicide or oil control (glyphosate 10 mg kg^−1^ or pongamia oil PO 50 mM). Some morphological features, non-enzymatic and enzymatic antioxidants, and gene expression were observed. Glyphosate-treated plants sprayed with PO 50 mM (GLY + PO 50) showed increased root biomass (0.28 g-*p* ≤ 0.001), shoot biomass (1.2 g-*p* ≤ 0.01), H_2_O_2_ (68 nmol/g), and the activities of superoxide dismutase (SOD; 40 mg-*p* ≤ 0.001), catalase (CAT; 81.21 mg-*p* ≤ 0.05), ascorbate peroxidase (APX; 80 mg-*p* ≤ 0.01) and glutathione reductase (GR; 53 min/mg-F_4,20_ = 15.88, *p* ≤ 0.05). In contrast, these plants showed reduced contents of Malondialdehyde (MDA; 30 nmol/g-*F*_4,20_ = 18.55, *p* ≤ 0.01), O_2_ (0.6 Abs/g), Prolne (Pro; 345 µg/g), Glutathine (GSH; 341 nmol/mg-*p* ≤ 0.001), ascorbate (AsA; 1.8 µmol/gm), ascorbic acid (AA; 1.62 mg-*p* ≤ 0.05) and dehydroascorbate (DHAR; 0.32 mg *p* ≤ 0.05). The gene expression analysis was conducted for seven oxidative stress related genes besides the house-keeping gene Actin as a reference. The gene *CYP1A1450* showed the highest mRNA expression level (6.8 fold ± 0.4) in GLY-treated tomato plants, whereas GLY-treated plants + PO 50 showed 2.9 fold. The study concluded that foliar spray of 50 mM pongamia oil alleviated the toxic effects of glyphosate on tomato plants in the form of increased root and shoot biomass, SOD, CAT, APX, and GR activity, while reduced MDA, O_2_, Pro, GSH, AsA, AA, DHAR, and gene *CYP1A1450* expression.

## 1. Introduction

For successful and high yield crop production, weeds have to be controlled, but the centuries-old practice of hand weeding is no longer sustainable due to a shortage of workers, because people move from rural to urban areas. Therefore, the use of herbicides will remain unavoidable and it will be the main factor in sustainable crop production in the near future [1].

Glyphosate [N-(phosphonomethyl) glycine CAS#1071-83-6] is the most used and studied broad-spectrum organophosphorus herbicide worldwide [2]. It is a widely applied herbicide against perennial and annual weeds in agriculture, silviculture, domestic gardens, and urban areas [3].

Physiologically, glyphosate inhibits the 5-enolpyruvylshikimate-3-phosphate synthase (EPSPS) enzyme from the shikimate pathway that prevents the biosynthesis of the amino acids phenylalanine, tyrosine, and tryptophan [4]. However, glyphosate has also indirect effects on plant physiology such as its role as a metal chelator of minerals that are important co-factors for enzymes, biomolecular constituents, anti-oxidative systems as well as crop rhizosphere microbiota. It also increases reactive oxygen species (ROS) production, which negatively affects photosynthesis by decreasing chlorophyll pigment, photochemical efficiency, and C metabolism, leading to a reduction in plant growth and production [5,6,7].

There was a notion that resistance to glyphosate was unlikely to evolve due to its transition-state simulation, absence of known active herbicides’ transporters in plants, and lack of plant metabolism. Indeed, glyphosate was used primarily as a non-selective herbicide before/after planting. Glyphosate-resistant weeds (GRWs) were not found during the first 15 years of its use (1972–1997), but 220 weed species have evolved resistance to one or more herbicides among them 404 unique cases, species versus site of action, globally [8,9]. GRWs may adopt different mechanisms such as exclusion by vacuolar sequestration, reduced translocation through rapid leaves necrosis, molecular mechanisms, and inheritance of EPSPS gene duplication and EPSPS target-site mutations [10].

To reduce glyphosate detrimental effects on crops, many strategies have been adopted. The most successful is the use of glyphosate-resistant crops (GRCs). The main GRCs include soybeans [*Glycine max* (L.) Merr.], cotton (*Gossypium hirsutum* L.), corn (*Zea mays* L.), alfalfa (*Medicago sativa* L.), canola (*Brassica napa* L.), and sugar beet (*Beta vulgaris* L.). These genetically modified herbicide-resistant crops (HRCs) relied on the impact of just one gene, *cp4 epsps* that encodes for glyphosate-resistant 5-enolpyruvylshikimate-3-phosphate synthase or EPSPS [11].

Other alternatives to alleviate glyphosate damaging effects on crop plants were adopted. For example, silicon or nano-SiO_2_ co-application was reported to reduce glyphosate induced oxidative stress in crops. Silicon or nano-SiO_2_ prevents most of the glyphosate phytotoxic effects on growth, removes ROS, and upregulates the main antioxidant enzymes, including SOD, CAT, and APX [12]. Foliar spray of Salicylic acid (SA) was recommended to alleviate the stress of glyphosate on soybean plants [13]. Glyphosate induced toxicity and stress were also alleviated in chickpea plants by bacteria (*Burkholderia cepacia*), which is herbicide tolerant, phosphate solubilizing and carry plant growth promoting activities [14]. Foliar Ni application was applied to ameliorate glyphosate drift injury to *Triticum durum* or wheat [15].

*Pongamia pinnata* (L.) plant (Family: Fabaceae) is a medium-sized glabrous semi-evergreen indigenous tree, occurring in Southeast Asia and the Indian subcontinent. It is a fast-growing, drought and salt tolerant plant that produces seeds with high contents of non-edible oil, which can be used as a renewable energy source [16]. *P.pinnata* seeds contain 20–42% oil of their dry weight [17]. The dominant fatty acids of *P. pinnata* seed oil are palmitic acid, stearic acid, linoleic acid, and eicosenoic acid [16]. *P. pinnata* seed oil, which contains karanjin, a furano-flavonoid compound, was found effective as an insecticidal and larvicidal, but its application for agricultural pest control is limited by its low aqueous solubility, high photosensitivity, and high volatility [18]. Pongamia compost improved the yield and influenced the growth after 35 days of tomato transplants [19]. *P. pinnata* seed ethyl acetate extract showed a potent antimicrobial, antibiofilm, antioxidant, and anticancer agent [20]. *P. pinnata* seeds also contain pongamol, a flavonoid active agent, which exhibits diverse pharmacological activities [21]. These findings encouraged us to evaluate the pongamia oil properties on tomato oxidative stress and antioxidant response against glyphosate treatment.

The objective of this study was to investigate the protective effects of pongamia oil against glyphosate treatment on tomato (*S. lycopersicum*) plants. Some morphological, antioxidant, and mRNA of some metabolic gene expressions were analyzed to determine the ability of pongamia oil to alleviate glyphosate toxicity through these markers in tomato plants.

## 2. Results

### 2.1. Root Biomass Estimation

**Figure 1:**
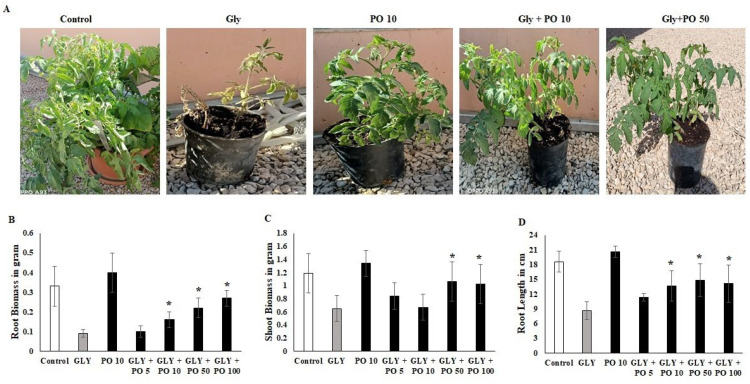
(**A**) Tomato plants untreated (Control) or treated with GLY, PO 50, or a mix of both (GLY + PO 10; GLY + PO 50). (**B**) Root Biomass. (**C**) Shoot biomass. (**D**) Root length. Data presented are mean ± SD (*n* ≥ 4). * indicate significant statistical differences between treatments and GLY-treated plants (Tukey’s test: *p* ≤ 0.05). Black bars indicate the treatment with PO and GLY + PO, the grey bar indicate GLY treatment and the white bar indicates the untreated control.

Treatment of *S. lycopersicum* with GLY showed significantly reduced root biomass growth compared to other treatments and the control (0.1 g-*p* ≤ 0.001). In stark contrast, foliar spraying of 50 mM pongamia oil (PO 50) displayed significant root biomass growth (0.4 g-*F*_4,20_ = 21.11, *p* ≤ 0.001). GLY + PO treatments displayed solid root biomass growth in a dose dependent manner against *S. lycopersicum* (Figure 1). Moreover, there is no significant difference between the combination treatment of GLY + PO 100 (0.32 g-*p* ≤ 0.001) and GLY + PO 50 (0.28 g-*p* ≤ 0.001) (Figure 1B).

### 2.2. Shoot Biomass Estimation

Treatment of *S. lycopersicum* with GLY showed a reduced shoot biomass growth compared to other treatments and the control (1.1 g-*p* ≤ 0.001), while spraying of tomato plants with PO 50 induced higher shoot biomass growth (1.4 g-F_4,20_ = 14.88, *p* ≤ 0.001) compared to other treatments, but there is no significant difference from the control (*p* ≤ 0.001). Correspondingly, GLY + PO 100 (1.0 g-*p* ≤ 0.001) and GLY + PO 50 (1.2 g-*p* ≤ 0.001) showed higher shoot biomass as compared to GLY + PO 5 (0.8 g-*p* ≤ 0.001) and GLY + PO 10 (0.6 g-*p* ≤ 0.001) (Figure 1C).

### 2.3. Root Length Estimation

Treatment of *S. lycopersicum* with GLY showed significantly reduced root length compared to other treatments and the control (15 cm-*p* ≤ 0.001), which was in contrast to treatment with PO 50 that induced a higher ratio of 21 cm (*F*_4,20_ = 27.21, *p* ≤ 0.001) compared to other dosages and the control. Even though, there is no significant difference between GLY + PO 10 (13 cm-*p* ≤ 0.001), GLY + PO 50 (15 cm-*p* ≤ 0.001) and GLY + PO 100 (14 cm-*p* ≤ 0.001) (Figure 1D).

### 2.4. Estimation of MDA, H_2_O_2_ and O_2_

**Figure 2:**
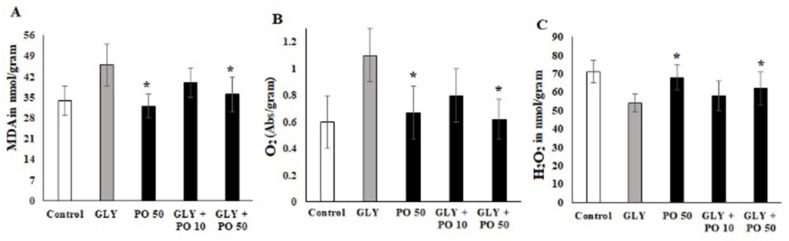
Tomato plants untreated (Control) or treated with GLY, PO 50, or a mix of both (GLY + PO 10; GLY + PO 50). (**A**) MDA, (**B**) O_2_ and (**C**) H_2_O_2_ concentration. Data presented are mean ± SD (*n* ≥ 4). * indicate significant statistical differences between treatments and GLY-treated plants (Tukey’s test: *p* ≤ 0.05). Black bars indicate the treatment with PO and GLY + PO, the grey bar indicate GLY treatment and the white bar indicates the untreated control.

GLY induced higher MDA (46 nmol/g; *F*_4,20_ = 18.55, *p* ≤ 0.001) compared to PO 50 (30 nmol/g-*F*_4,20_ = 18.55, *p* ≤ 0.001), GLY + PO 10 (41 nmol/g-F_4,20_ = 18.55, *p* ≤ 0.001), GLY + PO 50 (35 nmol/g-F_4,20_ = 18.55, *p* ≤ 0.001) and the control (33 nmol/g-F_4,20_ = 18.55, *p* ≤ 0.001) (Figure 2A). GLY also induced a higher level of O_2_ (1.1 Abs/g) compared to PO 50 (0.7 Abs/g), GLY + PO 10 (0.8 Abs/g), GLY + PO 50 and the control (0.6 Abs/g) (Figure 2B). Opposite to MDA and O_2_, GLY reduced H_2_O_2_ (54 nmol/g) compared to the control (70 nmol/g), but treatment with PO 50 slightly uplifted the level of H_2_O_2_ (68 nmol/g) as well as GLY + PO 50 (60 nmol/g) and GLY + PO 10 (58 nmol/g) (Figure 2C).

### 2.5. Quantification of Non-Enzymatic Markers

**Figure 3:**
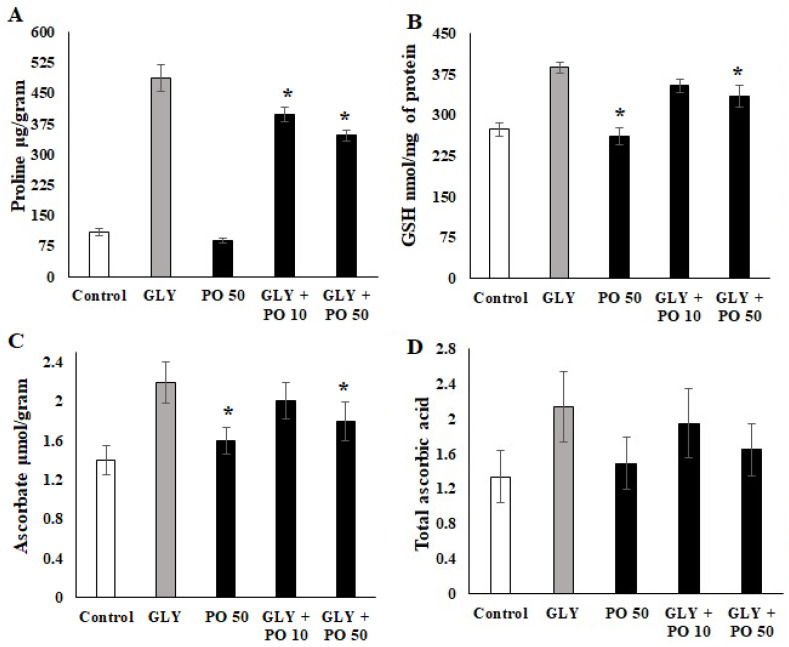
Tomato plants untreated (Control) or treated with GLY, PO 50, or a mix of both (GLY + PO 10; GLY + PO 50). (**A**) Proline, (**B**) GSH (**C**) Ascorbate and (**D**) Ascrobic Acid concentration. Data presented are mean ± SD (*n* ≥ 4). * indicate significant statistical differences between treatments and GLY-treated plants (Tukey’s test: *p* ≤ 0.05). Black bars indicate the treatment with PO and GLY + PO, the grey bar indicate GLY treatment and the white bar indicates the untreated control.

GLY treatment significantly increased (*F*_4,20_ = 28.12, *p* ≤ 0.001) the level of proline (465 µg/g) in *S. lycopersicum* compared to the control (78 µg/g) and sprayed PO 50 (73 µg/g). The combination of GLY + PO 10 increased the proline rate (383 µg/g) as well as GLY + PO 50 (345 µg/g) (Figure 3A). GLY treatment also significantly increased GSH (378 nmol/mg) level (*F*_4,20_ = 28.12, *p* ≤ 0.001) compared to the control (288 nmol/mg-*p* ≤ 0.001), and other treatments; PO 50 (265 nmol//mg-*p* ≤ 0.001), GLY + PO 10 (365 nmol/mg-*p* ≤ 0.001), and GLY + PO 50 (341 nmol/mg-*p* ≤ 0.001) (Figure 3B). In parallel, the ascorbate level was also increased by GLY treatment (1.92 µmol/g; *F*_4,20_ = 21.12, *p* ≤ 0.001) compared to the control and other treatments (Figure 3C). The level of total ascorbic acid increased in the GLY treatment (2.2 mg; *F*_4,20_ = 18.22, *p* ≤ 0.001) compared to the control (1.3 mg-*p* ≤ 0.001), PO 50 (1.4 mg-*p* ≤ 0.001), GLY + PO 10 (1.8 mg-*p* ≤ 0.001), and GLY + PO 50 (1.62 mg-*p* ≤ 0.001) (Figure 3D).

### 2.6. Quantification of Enzymatic Markers

**Figure 4:**
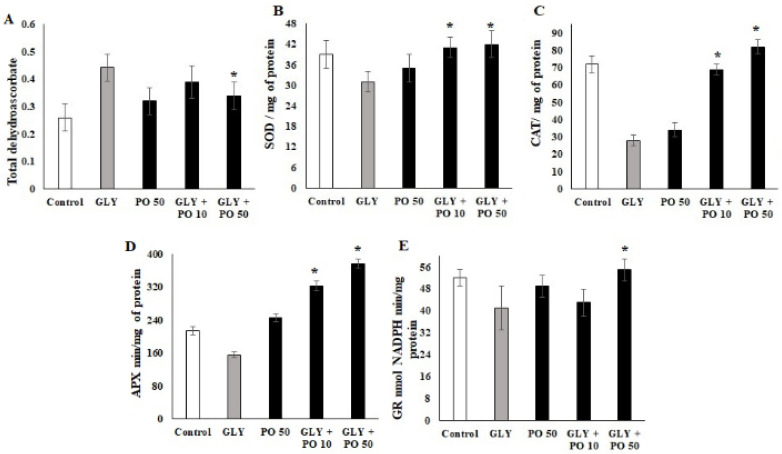
Tomato plants untreated (Control) or treated with GLY, PO 50, or a mix of both (GLY + PO 10; GLY + PO 50). (**A**) total dehydroascorbate, (**B**) SOD, (**C**) CAT, (**D**) APX and (**E**) GR concentration. Data presented are mean ± SD (*n* ≥ 4). * indicate significant statistical differences between treatments and GLY-treated plants (Tukey’s test: *p* ≤ 0.05). Black bars indicate the treatment with PO and GLY + PO, the grey bar indicate GLY treatment and the white bar indicates the untreated control.

The level of dehydroascorbate was also significantly elevated by the GLY treatment (0.46 mg; *F*_4,20_ = 18.22, *p* ≤ 0.001) compared with other treatments and the control (Figure 4A). In contrast, the level of SOD was decreased by GLY treatment (30.21 mg-F_4,20_ = 12.19, *p* ≤ 0.001) compared to the control (38 mg-*p* ≤ 0.001), PO 50 (33 mg-*p* ≤ 0.001), GLY + PO 10 (40 mg-*p* ≤ 0.001), and GLY + PO 50 (39 mg-*p* ≤ 0.001) (Figure 4B). Also, the level of CAT was significantly decreased by the GLY treatment (24 mg-F_4,20_ = 12.19, *p* ≤ 0.001) compared to the control (70 mg-*p* ≤ 0.001), but it was reversed by PO 50 (32 mg-*p* ≤ 0.001), GLY + PO 10 (68.2 mg-*p* ≤ 0.001), and GLY + PO 50 (81.21 mg-*p* ≤ 0.001) in a dose dependent manner (Figure 4C). A similar trend was observed in the APX level where GLY treatment displayed a significantly reduced protein rate (23 mg-F_4,20_ = 17.22, *p* ≤ 0.001) compared to the control, while the combination of GLY + PO 50 (80 mg-*p* ≤ 0.001) showed higher protein rate compared to other treatments and the control (Figure 4D). Correspondingly, the level of NADPH level was uplifted by the GLY + PO 50 treatment (53 min/mg-F_4,20_ = 15.88, *p* ≤ 0.001) compared to other treatments (Figure 4E).

### 2.7. Effect of PO on Antioxidant and Metabolic mRNA Markers in S. lycopersicum

**Figure 5:**
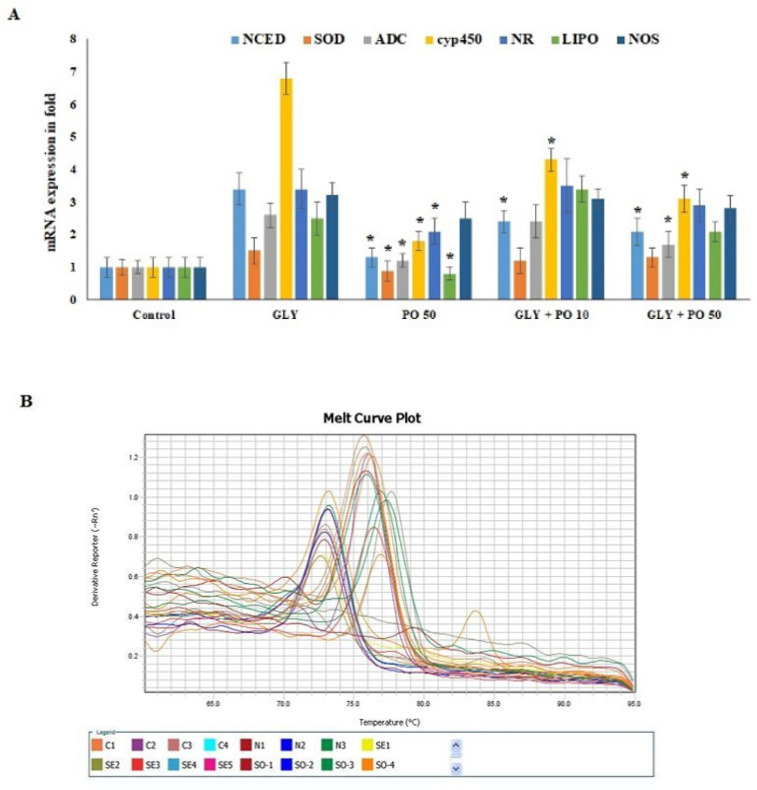
(**A**) mRNA expression in tomato plants untreated (Control) or treated with Gly, pongamia oil, or a mix of both (Gly + PO 10; Gly + PO 50). (**B**) Melting curve plot for mRNA expression of *NCED*, *SOD*, *ADC*, *CRY1A1450*, *NR*, *LIPO* and *NOS* markers. Data presented are mean ± SD (*n* ≥ 4). * indicate significant statistical differences between treatments and GLY-treated plants (Tukey’s test: *p* ≤ 0.05).

*CYO1A1450* gene showed the highest mRNA expression level (6.8 fold ± 0.4) in *S. lycopersicum* plants that were exposed to glyphosate. Whereas tomato plants challenged with GLY + PO 10 or GLY + PO 50 showed 4.2 and 2.9 fold, respectively (Figure 5A). Compared to GLY-treated tomato plants, those treated with pongamia oil (PO 50) alone, the mRNA expression of 9-cis-epoxycarotenoid dioxygenase (*NCED*), superoxide dismutase (*SOD1*), arginine decarboxylase (*ADC*) and Lipooxygenase (*LIPO*) markers were not activated. On the other hand, cytochrome P 450 (*CYP1A1450*), nitrate reductase (*NR*), and nitric oxide reductase (*NOS*) were significantly activated by PO 50 treatment. The comparative study clearly explained that PO 50 with GLY exposure regulates the antioxidant and metabolic expression factors and is merely significant to non-treated groups (Figure 5A,B). mRNA expression of *NCED*, *ADC,* and *CYP1A1450* actively responded to treatment (GLY exposure showed 3.5, 2.9, and 6.6 compared to 2.3, 1.7, and 2.9 mRNA fold in GLY + PO 50-treated group) and opened a new treatment procedure to control herbicide tolerance in *S. lycopersicum*.

## 3. Discussion

Besides its ability to kill weeds by affecting the EPSPS synthase enzyme resulting in an accumulation of shikimic acid and disturbing the biosynthesis of aromatic amino acids [22], GLY causes several other toxic effects on plant physiology. These physiological effects include mineral nutrition, photosynthesis, plant hormones, and oxidative stress, which affect plant growth [6]. GLY could deprive plants of important nutrients due to its cation chelating ability caused by its carboxyl and phosphonate groups. It forms complexes with nutrients in plant tissues, which affect their biological processes such as photosynthesis [23]. It was found to decrease Mg content and N assimilation, which prevent chlorophyll biosynthesis and reduce chlorophyll content in plants leading to a decreased photosynthetic rate [23,24,25,26,27]. GLY also reduced C exchange and CO_2_ assimilation capacity, which affected photosynthesis [24,28]. GLY was found to reduce N uptake in plants by affecting N fixation and/or assimilation [26,29,30] and its interference with symbiotic bacteria [31]. GLY was reported to inhibit root-to-shoot translocation of micronutrients, i.e., Mn, Fe, Zn, and reduced shoot concentration of mineral nutrients, especially Ca, Mg, Fe, and Mn [32,33].

The metabolically active sites i.e., shoot and root apical meristems are the main production sites of plant hormones. GLY accumulation in these active sites may disturb plant hormonal balance, which may lead to growth and development abnormalities [23]. One of these hormones is Indole-3-acetic acid (IAA). This auxin is synthesized from tryptophan and indolic tryptophan precursor, which are products from the shikimic acid pathway. Therefore, GLY may prevent the IAA biosynthesis by inhibiting the shikimate pathway. It was observed that GLY affected the expression of genes involved in the auxin response factor, genes encoding proteins in the auxin-responsive family, and IAA genes, which may trouble cell enlargement and plant growth [34]. Cytokinin (Cyt) content were reduced in three and four-year-old *Picea pungens* when treated with a mixture of GLY and haxazinone [35]. GLY was also found to affect rhizospheric interactions between plants and microorganisms by interfering in the balance of IAA which may lead to lower root nodulation [36]. The harmful effects of GLY on maize plants were alleviated by phenylurea cytokinin 4PU-30 treatment [37]. GLY was also shown to interfere with other hormones such as ethylene [38] and abscisic acid (ABA) [34].

Some or all of these GLY effects could explain the reduced plant root and shoot growth that was observed in this study and previous studies [12,39,40]. However, the application of PO alleviated those effects.

The antioxidant defense system of plants equilibrates between the production and reduction of reactive oxygen species (ROS) to eliminate its toxic effects [41]. Biosynthesis of enzymatic and non-enzymatic antioxidants is a mechanism developed by plants to cope with oxidative stress resulting from ROS accumulation. Activities of ROS-scavenging enzymes and the content of MDA are among the systems that are commonly used as indicators of oxidative stress in plants [42]. GLY inhibition of the shikimate pathway most probably causes oxidative stress in plants [43]. In this study, the level of lipid peroxidation or MDA, Pro content, GSH, H_2_O_2,_ and AsA were increased in GLY-treated tomato plants, but the foliar application of PO reversed these levels. These results are in line with the studies on maize leaves, *Handroanthus chrysotricus* (Mattos), and *Garcinia gardneriana* (Zappi), and wheat treated with GLY [22,37,40,44,45]. However, it was found that the treatment of maize with phenylurea cytokinin 4PU-30 protects it against GLY [37], while salicylic acid modulates wheat responses to GLY [40]. It was also found that aromatic amino acids could partially alleviate GLY-induced effects on growth of the *Faba Bean* and *Orobanche crenata* [46]. Foliar application of sodium nitroprusside was reported to boost *Solanum lycopersicum* tolerance to GLY [47] as well as silicon [12].

In this study, GLY-treated tomato plants expressed lower levels of SOD, CAT, APX, and GR than the normal plants (untreated plants), while the spray of PO increased the levels of these enzymes to that equal to or more than untreated plants. These results agree with a previous study [12] but contradict the studies in maize [37], rice [43], pea [48], duckweed [49], and wheat [40], where GLY increased the levels of these enzymes. Other pesticides were reported to increase SOD, CAT, and APX in tomato plants [50]. This could be attributed to metal deficiency caused by GLY such as Zn and Fe, which could impair the activities of these antioxidant enzymes [6]. GLY may Induces Fe deficiency that may block the biosynthesis of δ-aminolevulinic acid (ALA), which is a part of the chlorophyll biosynthesis pathway. CAT and peroxidase are enzymes involved in ALA biosynthesis and are highly sensitive to Fe deficiency [51]. Therefore, PO might have prevented these effects of GLY by freeing the metals from GLY and restoring the efficiency of oxidative enzymes.

PO contains saturated and unsaturated fatty acids, phenolic compounds including flavones; Karanjin and furanoflavone, chalcones; pongamol, obovatachalcone, and glabrachalcone, and many other miscellaneous compounds [52,53]. It was reported that the total phenolic contents of leaves and seeds exhibited scavenging and antioxidant abilities [54]. It was also reported that PO components are efficient multifunctional antioxidant compounds for protection against oxidative stress. Karanjin and pongapin have scavenging activities against nitric oxide, superoxide anion, total antioxidant activity, and iron chelating ability [55]. Therefore, it appears that some of these PO contents acted as antioxidants to alleviate the stress from tomato plants exposed to glyphosate.

There is evidence suggesting that weeds develop herbicide resistance by increasing the detoxification process of these herbicides through elevated levels of cytochrome P450 activity. Cytochrome P450 are a major group of plant enzymes, i.e., P450 monooxygenase (P450) superfamily, which mediate the oxidative degradation of a wide range of xenobiotic chemicals including herbicides. Cytochrome monooxigenase *CYTP450* transgenic tobacco plants showed higher PSII activity due to its higher ability to metabolize herbicides, which increased *CYP450* activity that lead to tolerance against oxidative stress after herbicide treatment [56,57]. This is in agreement with the results of this study, where the GLY-treated tomato plants showed higher expression of the *CYT1A1P450* gene, which was reduced by the foliar spray of PO.

These variations of mRNA expression regulated the defense factors and controlled the phytotoxic mediated mRNA expression

## 4. Materials and Methods

### 4.1. Plant Material and Growth Conditions

Seeds of *S. lycopersicum* L. cv. Micro-tom, obtained from a local florist in Al-Ahsa, Saudi Arabia, were used as biological material in the present work. Before sowing, seeds were surface disinfected with 70% (*v/v*) ethanol and 20% (*v/v*) sodium hypochlorite (5% active chloride), supplemented with 0.05% (*m/v*) Tween-20, for 5 min each, and subsequently washed several times with deionized water (dH_2_O). As a preliminary experiment to determine the acute toxicity dose of glyphosate, healthy 25 tomato seeds were placed in a growth chamber, under controlled conditions of temperature (25 °C), photoperiod (16 h light/8 h dark), and photosynthetic active radiation (120 µmol m^−2^ s^−1^). After 8 days, all the grown plantlets were transferred to plastic pots containing 200 g of pot soil with 100 mg kg^−1^ GLY. To ensure nutrient availability, 120 mL of modified Hoagland solution were added to a cup placed under each pot at the beginning of the assay. Afterward, dH_2_O was added when required, and plants were grown for 28 days in a growth chamber, as described above. Thereafter, the sub-lethal dose (10 mg kg^−1^ GLY) was considered for this study.

### 4.2. Experimental Design

In order to investigate the possible role of PO to alleviate GLY-induced toxicity in tomato (*S. lycopersicum*), experimental plantlets were divided into different experimental groups:

- Control; plants grown in OECD substrate (negative control).
- GLY; plants grown in OECD substrate contaminated with sub-lethal dose of 10 mg kg^−1^ GLY (positive herbicide control).
- PO 10; plants grown in OECD substrate treated once a week with 10 mM pongamia oil by foliar spraying (positive oil control).
- GLY + PO 5; plants grown in OECD substrate contaminated with sub-lethal dose of 10 mg kg^−1^ GLY and treated once a week with 5 mM pongamia oil by foliar spraying. This was later omitted, because the results were not significant from GLY + PO 10.
- GLY + PO 10; plants grown in OECD substrate contaminated with sub-lethal dose of 10 mg kg^−1^ GLY and treated once a week with 10 mM pongamia oil by foliar spraying.
- GLY + PO 50; plants grown in OECD substrate contaminated with sub-lethal dose of 10 mg kg^−1^ GLY and treated once a week with 50 mM pongamia oil by foliar spraying.
- GLY + PO 100; plants grown in OECD substrate contaminated with sub-lethal dose of 10 mg kg^−1^ GLY and treated once a week with 100 mM pongamia oil by foliar spraying. This was later omitted, because the results were not significant from GLY + PO 50.

The selection of pongamia oil concentrations was based on previous bibliographic records [18,20] and set as 5, 10, 50, and 100 mM. Pongamia oil was purchased from SRP global exports, India mart (Tamil nadu, India). The concentrations of PO were sprayed once a week on plantlets according to OECD guidelines for the testing of plant chemicals (www.oecd.org; accessed on 10 December 2021). Plantlets of the control and GLY experimental groups were sprayed weekly with dH_2_O only. After 28 days of growth, five plantlets from each treatment were randomly selected, collected and immediately used for biometric analysis. In parallel, shoots and roots of five plantlets from each treatment were frozen in liquid nitrogen (N_2_) and stored at −80 °C for later use. For all studied parameters, including all biochemical procedures, at least four replicates of samples were used for each experimental group.

### 4.3. Quantification of Non-Enzymatic Antioxidants—Proline (Pro), Glutathione (GSH), Ascorbate (AsA) and Total Ascorbic Acid

Pro was quantified in frozen plantlet samples by the ninhydrin-based colorimetric assay [12] and measured absorbance at 520 nm. The levels of Pro were determined based on a linear calibration curve that was obtained with solutions of known concentration, and results were expressed in mg g^−1^ fm (fresh mass). The quantification of GSH was accomplished by following the procedure described in [12]. Ascorbate (AsA) was quantified by spectrophotometry at 525 nm, based on the 2,2′-bipyridyl method [58]. Ascorbic acid (AA) in plantlets tissues (1 g fresh weight) was extracted with 20% trichloroacetic acid, which was then measured by a titrimetric method using 2, 6-dichlorophenol indophenol dye.

### 4.4. Measurement of Physiological Indexes of Enzymatic Antioxidants

To investigate the stress of glyphosate on tomato plantlets, the leaf tissue of GLY exposed plantlets was crushed using a chilled pestle and mortar kept in an ice bath. The crushed leaf tissue was homogenized in 200 mM, pH 6.0 phosphate buffer. The homogenate was centrifuged at 12,000× *g* for 20 min at 4 °C. The supernatant was stored at 4 °C and used for assays within 4 h. Enzymes activities were measured using previous methods [59]. One U of POD means an absorbance change of 0.01 U/min^−1^.

The enzyme activities of superoxide dismutase (SOD; EC1.15.1.1), catalase (CAT; EC 1.11.1.6), ascorbate peroxidase (APX; EC 1.11.1.11), glutathione reductase (GR; EC 1.8.1.7), and dehydroascorbate reductase (DHAR; EC 1.8.5.1) were measured. In the case of SOD, an aliquot of the supernatant was mixed with 10 µM sodium nitrate (NaNO_3_). Total SOD activity was performed based on the inhibition of the photoreduction of 2-nitrobenzoic acid (NBT) and measured by spectrophotometry at 560 nm [60]. For each sample, an appropriate volume of extract (30 µg of protein) was added to a reaction mixture containing 100 mM potassium phosphate buffer (pH 7.8), 0.093 mM EDTA, 12.05 mM L-methionine, 0.0695 mM NBT and 0.0067 mM riboflavin in a final volume of 3 mL. The enzymatic reaction was started by adding the riboflavin to the tubes, which were immediately placed under six fluorescent 8 W lamps for 10 min. After this period, the light source was removed in order to stop the reaction. Enzyme activity was expressed as units SOD mg^−1^ protein, in which one unit represents the amount of SOD required to inhibit NBT photoreduction by 50%. The evaluation of CAT and APX activity was accomplished by enzyme kinetics, by measuring the decomposition of H_2_O_2_ (ε240 nm = 39.4 M^−1^ cm^−1^) and AsA (ε290 nm = 2.8 mM^−1^ cm^−1^) over 2 min [61]. In both cases, the reaction was started by the addition of H_2_O_2_. Regarding DHAR and GR, changes in Abs at 265 and 340 nm were monitored to follow AsA (ε265 nm = 14 mM^−1^ cm^−1^) production and NADPH consumption (ε340 nm = 6.22 mM^−1^ cm^−1^), respectively. Results are expressed as µmol min^−1^ mg^−1^ protein. The original protocol was adapted to UV microplates, based on the optimization of Murshed [62].

### 4.5. H_2_O_2_ and Malondialdehyde (MDA) Content

The concentration of H_2_O_2_ was measured using a hydrogen peroxide assay kit (Cayman Chemicals, Ann Arbor, MI, USA), according to the manufacturer’s instructions. Absorbance values were measured at 570 nm using a Bio-Rad microplate reader and converted into concentrations based on standard curve data. 

The level of lipid peroxidation was determined by measuring the MDA content using the thiobarbituric acid test, as described previously [63]. Fresh leaf (0.1 g) was homogenized in 1 mL of 10% (*w/v*) trichloroacetic acid. The homogenate was centrifuged at 10,000× *g* for 10 min and the supernatant was collected, mixed with 0.67% (*w/v*) thiobarbituric acid (TBA), and incubated at 95 °C for 30 min on a heat block. It was quickly cooled on ice and finally centrifuged at 10,000× *g* for 10 min. Absorbance values of the supernatant were measured at 450, 532, and 600 nm and converted to concentrations (nM g^−1^ fresh weight) using the following equation: MDA content = 6.453 (A532–A600) − 0.563 (A450).

### 4.6. Estimation of O_2_ (ROS) Level in Shoot and Root of Tomato

ROS is a fundamental cellular signaling molecule capable of regulating various metabolic pathways. The amounts of ROS (O_2_) were determined spectrofluorometrically. Briefly, 1 g of shoot of each treatment was crushed in liquid nitrogen, and homogenized in darkness in 2 mL of lysis buffer (1 mM EDTA, 2 mM DTT, 1 mM PMSF, 0.2% Triton X-100, and 50 mM Tris-HCl; pH 7.4) and centrifuged at 12,000 rpm for 15 min at 4 °C. Supernatant was used to quantify the O_2_ level in samples using chromophore substrate (TMB). 1 mM Tris was used as a negative control. The reaction mixture was incubated at 37 °C for 1 h, and the excited color was measured in a spectrophotometer at 510 nm [64]. The level of O_2_ was expressed in arbitrary units (AU) per μg protein.

### 4.7. Gene Expression

The oxidative damage and stress tolerance of GLY- and PO-treated *S. lycopersicum* were quantified based on mRNA markers using real time PCR method. Leaf tissue (100 mg) was homogenized with liquid nitrogen and total RNA was extracted using Trizol reagent (Invitrogen, San Diego, CA, USA) according to the manufacturer’s protocol. First-strand cDNA was synthesized following the instructions of the Super Smart cDNA Synthesis Kit (Takara, Otsu, Japan). Real-time quantitative PCR was performed using SYBR Green I master mix (Takara, Otsu, Japan) in a Bio-Rad iCycler iQ5 fluorescence real-time PCR system (Bio-Rad, Hercules, CA, USA). The annealing temperature of the target genes was optimized to 60 °C. The primers for 9-cis-epoxycarotenoid dioxygenase (*NCED*), superoxide dismutase (*SOD1*), arginine decarboxylase (*ADC*), cytochrome P 450 (*CYP1A1450*), nitrate reductase (*NR*), Lipooxygenase (*LIPO*), nitric oxide reductase (*NOS*) and actin genes are provided in Table 1. To normalize results, the Cq (quantification cycle value) was calculated as the relative expression level of each target gene and the housekeeping gene (Actin) in each sample [65].

### 4.8. Statistics

The data are expressed as mean ± SD. Statistical analyses were performed using GraphPad Prism 8.0 (GraphPad Software version 6.04 for Windows, La Jolla, California USA, www.graphpad.com). Statistical significance was determined by student *t*-test and one-way ANOVA with Tukey’s test for multiple-group comparisons. The level of significance was set at *p* < 0.05.

## 5. Conclusions

Glyphosate is an exceedingly valuable broad-spectrum herbicide and it is the sole herbicide that targets the EPSPS enzyme. Ever since glyphosate became a generic compound, its cost has dropped dramatically, and farmers are relying more on it all over the world. In this study, the sensitivity of glyphosate application with/without pongamia oil on tomato saplings much differed. The physiological response indices such as MDA content, O_2_, Pro, GSH, Ascorbate, Ascorbic acid, and dehydroascorbate were higher after glyphosate exposure, but they were recovered to their normal or higher levels by foliar spray of pongamia oil. Glyphosate application also reduced the activities of antioxidant enzymes that were tested, i.e., SOD, CAT, APX, and GR, but after treatment of tomato saplings with pongamia oil, there were significant effects on these anti-oxidant enzymes. These results show that more studies are required to investigate and minimize the effects of glyphosate by foliar spray of pongamia oil on tomato plants or other crops, which we plan to do in the future. However, since there are already different commercial formulations incorporating pongamia oil in the agroindustry, it can be concluded that pongamia oil may represent a promising tool to overcome the side effects of glyphosate on crop plants in a real agricultural context.

## Figures and Tables

**Table 1:** Names and sequences of primers used in RT-PCR and PCR product length in base pairs (bp).

Primer Name	Forward Sequence	Reverse Sequence	PCR Product (bp)
NCED	GAA CTT CGT CGT CAT TCC TG	CAT CTT TCG CGT ACT TAT CCA	197
SOD1	ACC ACA ACC AGC ACT ACC AAT	GTC CAG GAG CAA GTC CAG TTA	186
ADC	TGC TTG AAG TGT CTC TTG	GAT TGC GGT CAT AAC ATA AG	213
*CYP1A1450*	GGG ACA TTA TTC GGG AGA GG	GAT GAT GAA AGC CGA TGA CAG	167
NR	ATC ACC CAG AGA AGC CAA CA	GAG GGT CTC ATC GGT AGC TC	224
LIPO	GCC TCT CTT CTT GAT GGA	GTA GTG AGC CAC TTC TCC AA	178
NOS	GAG CTC CGT TAC ACA CAT CG	CGA CAC CGT CCA CAA AGA AT	211
Actin	GAG AAG CAC ATT CCC TGA AAG	AGA ACT CCA CCA TCA CCA CC	203

## Data Availability

Not applicable.
